# Reversibility and criticality in amorphous solids

**DOI:** 10.1038/ncomms9805

**Published:** 2015-11-13

**Authors:** Ido Regev, John Weber, Charles Reichhardt, Karin A. Dahmen, Turab Lookman

**Affiliations:** 1 School of Engineering and Applied Sciences, Harvard University, 29 Oxford Street, Cambridge, Massachusetts 02138, USA; 2 Center for Nonlinear Studies and Theoretical Division, Los Alamos National Laboratory, Los Alamos, New Mexico 87545, USA; 3 Theoretical Division, Los Alamos National Laboratory, Los Alamos, New Mexico 87545, USA; 4 Department of Physics and Institute of Condensed Matter Theory, University of Illinois at Urbana Champaign, 1110 West Green Street, Urbana, 61801 Illinois, USA

## Abstract

The physical processes governing the onset of yield, where a material changes its shape permanently under external deformation, are not yet understood for amorphous solids that are intrinsically disordered. Here, using molecular dynamics simulations and mean-field theory, we show that at a critical strain amplitude the sizes of clusters of atoms undergoing cooperative rearrangements of displacements (avalanches) diverges. We compare this non-equilibrium critical behaviour to the prevailing concept of a ‘front depinning' transition that has been used to describe steady-state avalanche behaviour in different materials. We explain why a depinning-like process can result in a transition from periodic to chaotic behaviour and why chaotic motion is not possible in pinned systems. These findings suggest that, at least for highly jammed amorphous systems, the irreversibility transition may be a side effect of depinning that occurs in systems where the disorder is not quenched.

## Abstract (web summary)

The onset of yield, where a material starts to deform irreversibly, remains poorly understood for amorphous materials. Here, the authors use computer simulations that reveal how depinning-like processes in amorphous materials can result in large cooperative displacements of atoms during yield and cause irreversible, chaotic behaviour.

## Graphical abstract

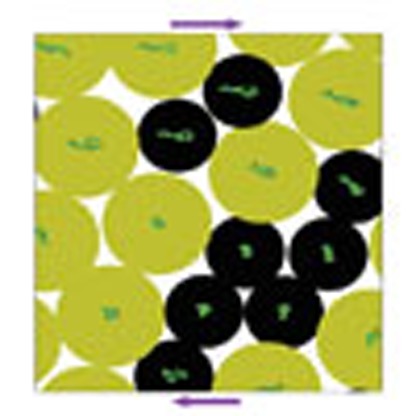

Amorphous solids such as plastics, window glass and amorphous metals are an important and ubiquitous form of matter. Industrial processing of such materials commonly involves plastic deformation. An outstanding issue in the amorphous plasticity community is defining and understanding yield^1^, the onset of irreversible behaviour, in terms of underlying universal processes. There is increasing evidence to suggest that the microscopic mechanism of plastic deformation is a local rearrangement of particles involving a change of nearest neighbours (so called shear transformation zones), which results in an Eshelby-like elastic field^2^,^3^,^4^,^5^. However, it was recently shown that these rearrangements can be repetitive or irreversible depending on the strain amplitude^6^,^7^,^8^,^9^,^10^,^11^,^12^,^13^,^14^,^15^,^16^,^17^,^18^ (a similar phenomenon has been observed in the shearing of colloidal suspensions, granular systems, dislocations and super-conducting vortices^19^,^20^,^21^,^22^,^23^,^24^,^25^,^26^,^27^,^28^ and in the compaction of granular matter^29^).

We have previously studied highly condensed amorphous solids (well above the jamming transition) under applied oscillatory shear and showed that for small strain amplitudes these systems evolve into periodic limit cycles during which particles change their (mechanical) equilibrium positions but follow the same trajectories for consecutive cycles (Fig. 1; ref. 6). Above a critical strain amplitude, the system does not settle into a limit cycle and the motion is chaotic with a positive maximal Lyapunov exponent^6^.

The transition from jammed to flowing behaviour in systems as diverse as earthquakes, charge density waves and disordered magnets is accompanied by the occurrence of avalanches of increasing sizes obeying power-law statistics, a signature of critical behaviour. In this work, we show using molecular dynamics simulations that in amorphous solids, at the same critical strain amplitude where irreversibility occurs, the system undergoes a non-equilibrium phase transition, which involves avalanches of diverging sizes. We analyse the avalanche statistics using a mean-field model for the depinning transition in plastic deformation that was used previously to describe plasticity in crystals and amorphous solids^30^,^31^. We show that large avalanches exist even below the transition, as was also observed in ref. 32, so that the existence of avalanches alone is not sufficient to explain the irreversible behaviour (see Fig. 2 for an example of the displacement field generated by an avalanche that is repeated periodically under repeated cycles of subcritical strain). However, we show that the cause of irreversible behaviour for strain amplitudes that are larger than a critical value is rooted in the changes of the energy landscape topology at depinning, which suggests why depinning and irreversibility occur at the same point.

## Results

### Simulations

**Figure 3:**
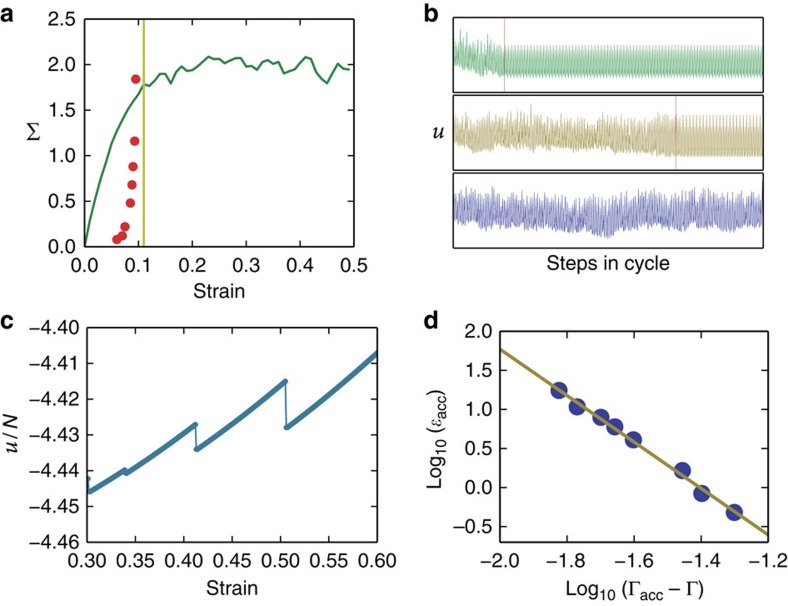
Transition to chaos at yield. (**a**) Stress–strain curve under linear shear (green line). The yellow line marks the transition to chaos and the red points show the number of cycles to reach a limit cycle under oscillatory shear. (**b**) Three different potential energy time series for three different maximal strain amplitudes growing from top to bottom. The red lines mark the onset of repetitive behaviour (limit cycle). (**c**) Typical behaviour of the potential energy per particle of an amorphous solid under quasi-static oscillatory shear. Large drops in the energy are separated by elastic regimes. The large drops correspond to rearrangements of particles. (**d**) Accumulated strain to reach a limit cycle as a function of the maximal strain amplitude minus the critical strain amplitude Γ_c_=0.11.

The data used in this work were generated using molecular dynamics simulations of systems of 1,024, 4,096 and 16,384 point particles in two dimensions interacting via an isotropic attractive–repulsive pairwise potential. In each case, we prepared an initial amorphous configuration and applied oscillatory athermal quasi-static shear while controlling the maximal strain amplitude in each run. The energy was kept at a minimum after each straining step using the Fast Inertial Relaxation Engine (FIRE) algorithm that reaches the steady state more quickly than standard overdamped dynamics algorithms (a more detailed discussion of the simulation methods can be found in the methods section and in refs 6, 33). In previous work^6^, we have shown that when the strain amplitude is increased, the system undergoes a transition from periodic to chaotic dynamics. In Fig. 3a, we show the point of transition with respect to a stress–strain curve obtained by applying a constant positive strain rate. The point of transition is marked as a yellow line and the time to reach steady state as red points. In Fig. 3b, we show the potential energy per particle for three different strain amplitudes (top—low amplitude, middle—medium and bottom—large amplitude). We observe that as the amplitude becomes larger, it takes longer to reach a periodic limit cycle and at a strain amplitude comparable to the yield strain (whose system size dependence will be discussed below), the typical time to reach a limit cycle diverges as a function of the strain amplitude (Fig. 3a,d). This indicates a kind of dynamical transition, whose nature is yet to be clarified. Below we suggest that this behaviour is a result of a non-equilibrium phase transition that is related to the well-studied front depinning universality class.

### Theoretical background

**Figure 4:**
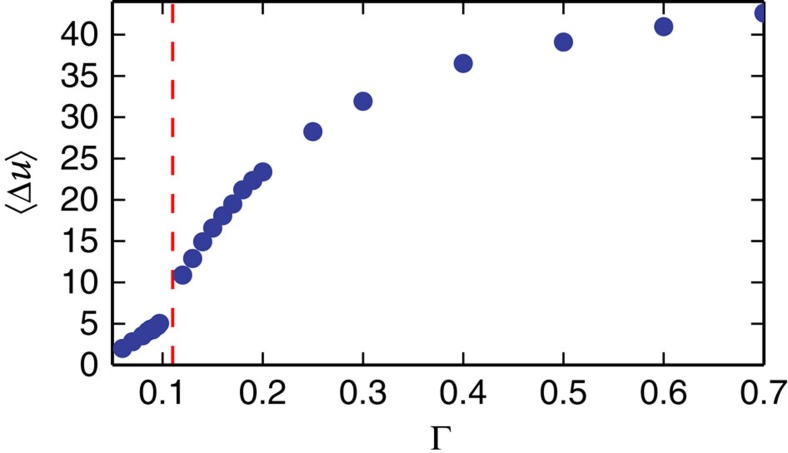
Mean energy drops. The mean energy drop 
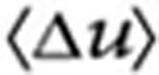
 as a function of the maximal strain amplitude Γ for the largest system size. Note the distinct cusp at the irreversibility point.

**Figure 5:**
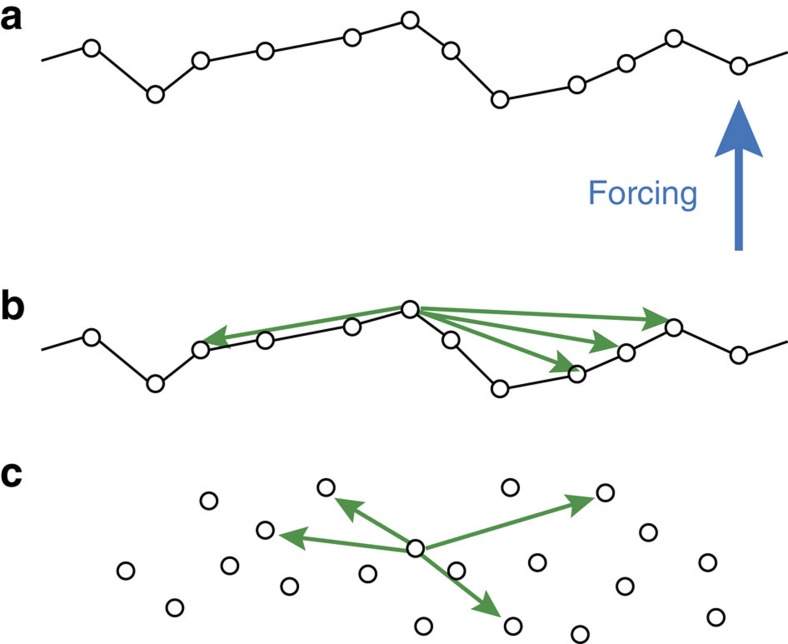
Depinning theory in the amorphous plasticity context. (**a**) Depinning theory describes the motion of an elastic interface (here a one-dimensional front) in a random potential. The circles represent the (plastic) displacement of each point in the front. The front is subject to an applied force that causes it to move but elements of the front are pinned locally and need to overcome energy barriers. The different elements of the front are connected by springs so that if one pinned site overcomes the energy barrier it is pulling its nearest neighbours (and only them). (**b**) If the interactions are long range, different pinned elements of the front interact with distant elements and the actual structure of the front becomes immaterial. (**c**) In this case, there is no real difference between the equations that describe a front and the equations that describe the interaction of some collection of pinning sites distributed in the material. A simple model of plasticity^31^, which belongs to the depinning universality class, has been shown to describe the dynamics of an amorphous solid under shear where ‘shear transformation zones' or ‘weak spots' are dispersed in the material and affect each other with long-range elastic interactions.

To understand the role of avalanches in the transition-to-chaos/yield, we studied the avalanche statistics for different maximal strain amplitudes and system sizes. Since the simulations were athermal, we identified drops in the potential energy (Fig. 3c) with plastic rearrangement events. For each simulation, we extracted all of the energy drops in the last shear cycle (to avoid transient effects), created a histogram of the energy drops and calculated the average energy drop 
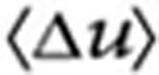
 for each maximal strain amplitude. We observe in Fig. 4 a cusp at the point at which the irreversibility transition occurs, followed by saturation to a value that depends on the system size, at large strain amplitudes. The cusp suggests that the irreversibility transition is related to a change in the avalanche dynamics, and the system size dependence of the saturation suggests that there is a saturating correlation length. To understand the avalanche statistics in this system, we invoke a simple model^31^ that belongs to the same universality class as the theory of front depinning (but with long-range interactions), which was originally developed to explain the motion of an interface in random media. This motion involves parts of an interface overcoming local energy barriers due to pinning sites and neighbouring locations in the interface ‘pulling' the site back. The forward motion of the interface occurs in avalanches. In the case of long-range interactions, such as the ones that exist in elasto-plastic systems, the notion of a ‘front' becomes more abstract since sites that are far apart affect each other and the notion of locality becomes blurred (see Fig. 5 for illustration). This explains why the same equations can also describe avalanche behaviour associated with the plasticity of amorphous solids in which the dynamics involves overcoming random energy barriers and long-range interactions, even if an actual front may not exist. The equations of motion describing the time evolution of the plastic displacement field *u*(**r**,**t**) controlled by overdamped dynamics are^31^:


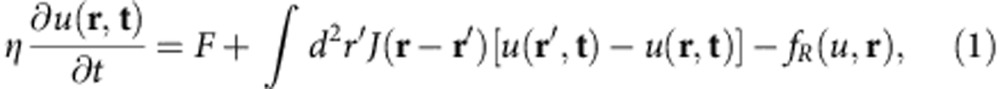


where *η* is the viscosity, *F* is an externally applied force, *r* is a position of a deformable region (Shear transformation zone), *J*(**r**−**r**′) is the Green's function for the elastic interaction between different ‘soft' regions located at points **r** and **r**′ and *f*_R_(*u*,**r**) is a random pinning potential representing the structural disorder inherent to such systems. This model assumes that the nature of the structure (the distribution of the random pinning forces *f*_R_(*u*,**r**)) does not change as a function of time. In amorphous solids, the randomness is self-generated and can (and typically does) change under plastic deformation. However, when the system is at a steady state under linear or cyclic shearing, one can assume that the disorder is fixed. Also, the scaling behaviour of the model predictions do not change if the pinning stresses randomly change in time. This model shows a non-equilibrium phase transition between a pinned, static state and a flowing state as the stress is slowly increased past a critical force *F*_c_ (ref. 31). The transition is a critical point involving correlated displacement jumps. These correlations are described in terms of a scaling theory, which was derived from a mean-field (infinite interaction range) approximation and renormalization group theory^30^,^31^. This theory was indeed shown to give a good description of the statistics of avalanches during plastic deformation in crystals^34^,^35^,^36^,^37^ and is now also being applied to amorphous solids^38^,^39^,^40^. For an applied external force, at zero velocity (quasi-static limit), it was found that at a critical force *F*_c_ the avalanche size distribution scales as:


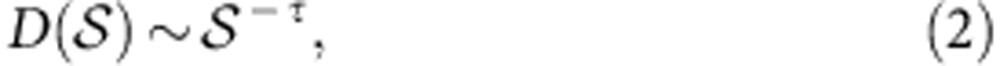


where *S* is the avalanche size and *τ* is a universal critical exponent. Below *F*_c_, the distribution follows the same power law but with a maximal size (cutoff):


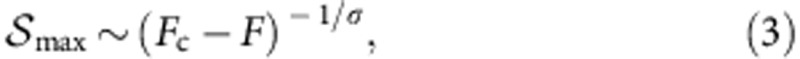


where *σ* is the cutoff exponent. Then, the distribution function takes the form:


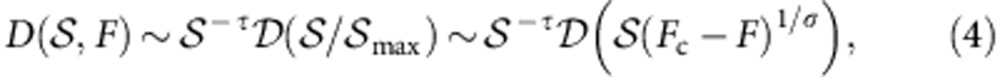


where 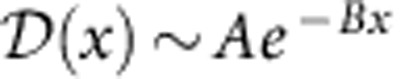 is a universal cutoff scaling function but the constants *A* and *B* are system specific^30^,^31^. Below we will see how depinning theory can help explain the observed changes in the avalanche statistics as a function of the maximal strain amplitude.

### Statistics under oscillatory shear

When applying the statistical theory of front depinning for amorphous solids under oscillatory shear, we have to modify the theory to take into account the different factors that were not included in the theory described above, which assumes a steady force. One issue is that the disorder in amorphous solids is not quenched, which can affect the statistics. For example, there could be weakening effects during an avalanche event, where the same site can be triggered more than once. This has been addressed by Dahmen *et al.*^31^ and was shown to affect the stress–strain curve but not the scaling exponents^30^. The second effect of having dynamic disorder is that the distribution that describes the random variable *f*_R_(*u*,**r**) can change during a cycle. We avoid this problem by performing statistics only for avalanches in ‘steady-state' cycles, when the avalanche statistics is stable. It is known that the exact distribution of the disorder does not affect the avalanche statistics so even if the disorder is different in different cycles, that should not change the scaling functions. Another issue is that the forcing is a ‘sawtooth', periodic strain profile. To take that into account, we have to rewrite equation (4) in terms of the strain and integrate over the different strain amplitudes. The relation between the stress and the strain shows hysteresis due to the nonlinear nature of plastic deformation (Supplementary Note 1; Supplementary Fig. 2). Since the forward and reverse straining branches of the hysteresis curve are statistically identical, we take into account only the forward direction. For the forward branch, we can model the relation between stress and strain using the scaling relation (Supplementary Note 1):


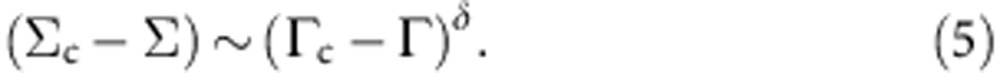


Where the critical strain Γ_c_ and critical stress Σ_c_ are related to the critical force *F*_c_, the shear modulus *μ* and the system size *L* by:


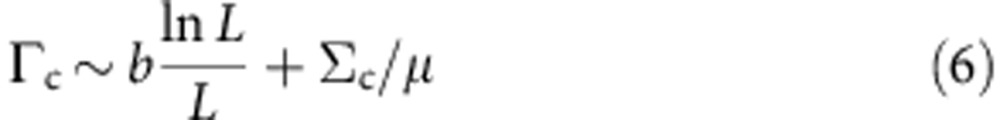


where *b* is a system-dependent constant. This expression is explained and verified in Supplementary Note 2 and demonstrated in Supplementary Fig. 3. By fitting to stress–strain curves in the steady state, we find that *δ*∼1.25 (Supplementary Note 1; Supplementary Fig. 4).

We substitute (equation 5) into equation (4) and obtain a scaling relation for the avalanche size distribution as a function of the strain amplitude:


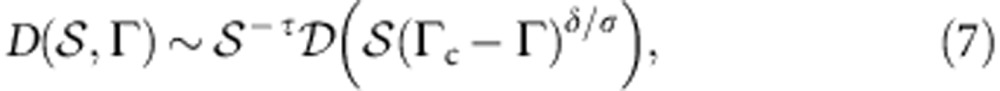


which would be expected to describe the avalanche statistics close to the critical strain amplitude. However, for oscillatory driving, the scaling function 
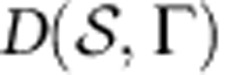
 requires corrections since the avalanche size distribution measured is a result of integration over a varying amount of applied strain. Since the strain increases and decreases periodically, the system spends time both below and above the critical strain amplitude. Because we are averaging over cycles, we need to integrate over the different strain amplitudes. We also simplify the calculations by making some approximations (Supplementary Note 3). Below the transition we get the equation:


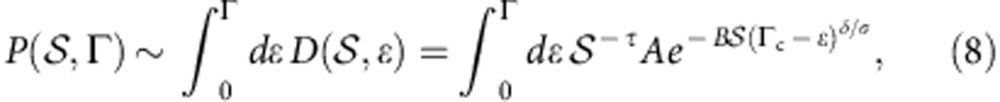


where 
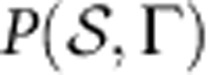
 is the distribution of avalanche sizes at maximal strain amplitude Γ, and *ɛ* is the instantaneous strain amplitude during a cycle *ɛ*∈[−Γ,Γ] (Supplementary Note 3 for a detailed derivation). If the maximal strain amplitude Γ is larger than the critical value, we have to average over the statistics both below and above the critical strain amplitude. Due to the quasi-static forcing (zero strain rate), for strains larger or equal to the critical strain amplitude, the system is expected to be exactly at criticality^41^, and the avalanche statistics is expected to behave as a pure power law:


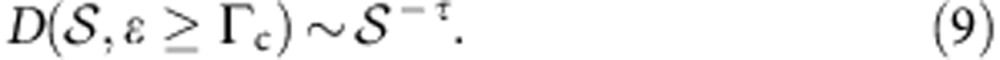


**Figure 6:**
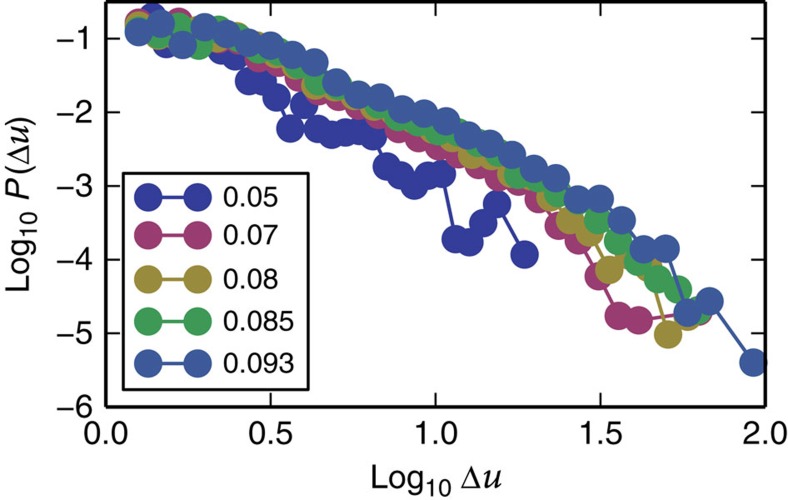
Fluctuations. Energy drop distribution generated from log histograms for five different maximal strain amplitudes below the transition for strain amplitudes Γ=0.05, 0.07, 0.08, 0.085 and 0.093.

**Figure 7:**
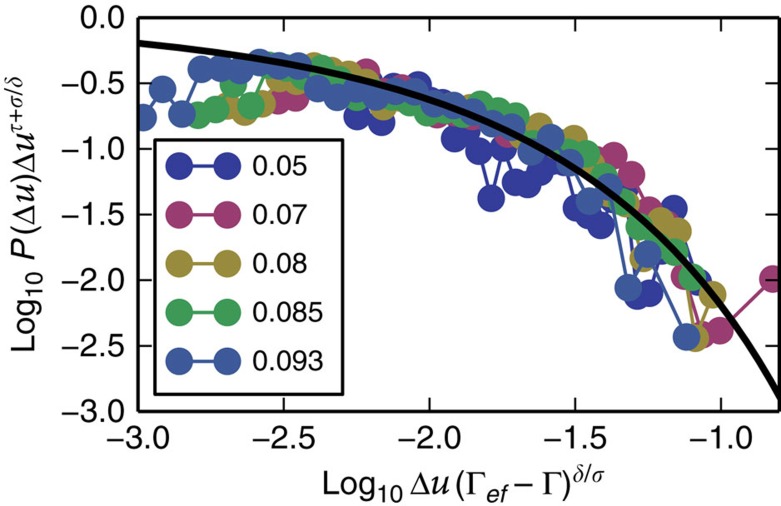
Data collapse. Data collapse for five different maximal strain amplitudes below the transition compared with the mean-field scaling function 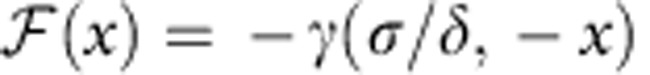, where *γ*(*a*,*x*) is the complementary gamma function (marked by a black line).

Substituting, we obtain:


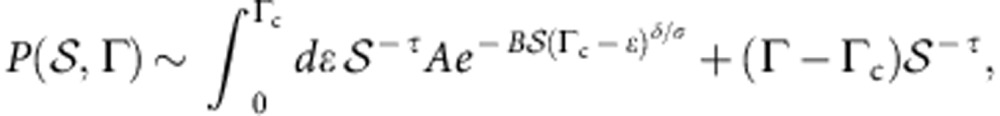


where we have performed the integral over the last term. As explained in the Supplementary Note 3, by changing the variable of integration in equation (8) we can obtain a scaling function for the fluctuations below the critical point (see Supplementary Note 3 for the derivation details):


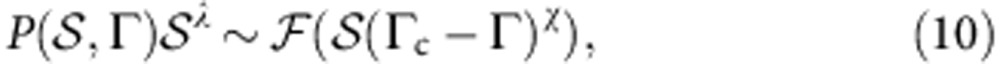


where *λ*=*τ*+*σ*/*δ* and *χ*=*δ*/*σ*. The scaling function is generally unknown. However, for mean field it was calculated to be 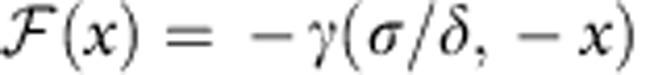, where *γ*(*a*,*x*) is the complementary gamma function and seems to agree with the data collapse (Figs 6 and 7; Supplementary Note 3). Avalanche sizes in plasticity are usually associated with the amount of slip, which is proportional to the stress drop. However, as was shown in refs 33, 42, in the steady state, the fluctuations of stress and potential energy drops are proportional due to a sum rule. We assume that to apply here as well (this is further explained and verified from the simulations data in the Supplementary Note 4 and Supplementary Fig. 5). Using equation (10), we find data collapses for five maximal strain amplitudes Γ=0.05, 0.07, 0.08, 0.085 and 0.093 at system size *N*=16,384 (Figs 6 and 7) from which we extract *λ* and *χ* (see Supplementary Note 5 for an explanation about the choice of Γ values). Since we have an estimate of *δ*, we can find the critical exponent values *τ*=1.04[0.26], *σ*=0.59[0.04]. The exponents deviate from the exponents found using mean-field theory, which are *τ*=1.5 and *σ*=0.5. However, a recent study by Salerno *et al.*^42^, for simulations under direct shear (not alternating), obtained different critical exponents for overdamped, underdamped and damped dynamics and the value of *τ* ranged between *τ*=1 (critically damped), *τ*=1.25 (overdamped) and *τ*=1.5 (underdamped) depending on the dynamics. Since the FIRE algorithm uses an inertia-like effect to minimize the energy, it is possible that inertial effects contribute to the deviation from mean-field theory, which was derived for overdamped dynamics (see Supplementary Note 6 and Supplementary Fig. 6 for a comparison of the algorithm relative to overdamped, underdamped and critically damped dynamics applied to a harmonic oscillator). Another possible reason for the deviation from mean-field exponents, which has been suggested recently, is the effect of anisotropic interactions^43^, at least for simulations in steady state, under linearly increasing shear. Below we use the critical exponents *τ* and *σ* to explain the observed cusp in the energy fluctuations (Fig. 4).

### Average fluctuations

**Figure 8:**
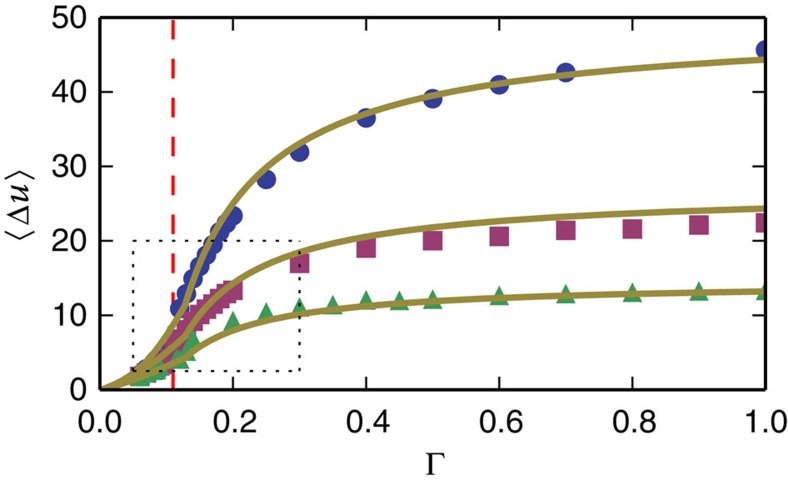
First moment. Average potential energy drops versus maximal strain amplitude for different system sizes: *N*=16,384(blue circles), *N*=4,096(purple squares), *N*=1,024(green triangles). The yellow lines are the theoretical results equation (11) where the integral was calculated numerically. The red-dashed line marks the transition to chaos point for *N*=16,384.

**Figure 9:**
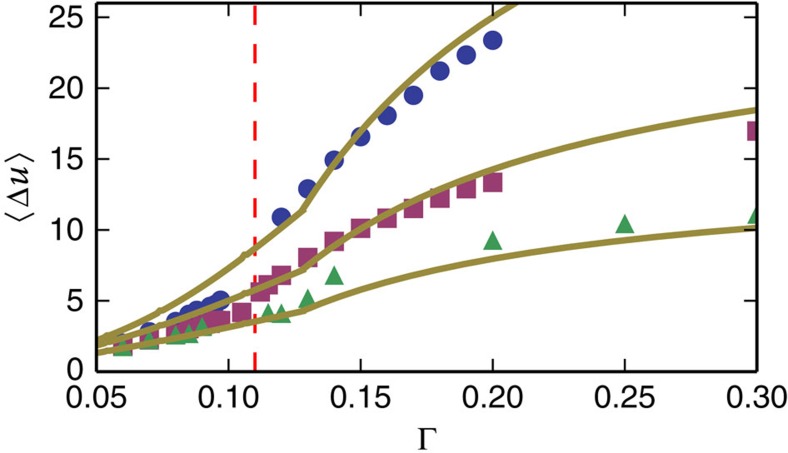
Transition point. Scaled up version of Fig. 8. Note the change in curvature at the critical points Γ_c_=0.115, 0.12 and 0.135 for *N*=16,384(blue circles), *N*=4,096(purple squares) and *N*=1,024(green triangles), respectively. Yellow lines are theoretical results.

From the relevant critical exponents, we can obtain the average avalanche size introduced in Fig. 4 using similar analysis as above (see Supplementary Note 7 for details):


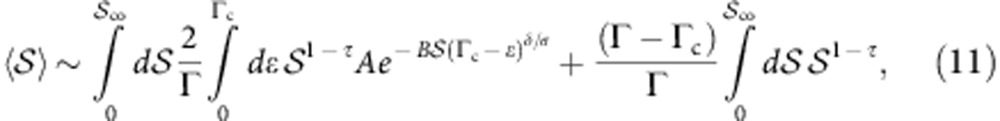


where 
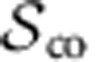
 is a cutoff avalanche size that depends on the system size in an unknown way, and we have divided the integral by Γ to perform a cycle average. For the critical exponents *τ* and *σ*, we used the values *τ*=1.04 and *σ*=0.59 that were obtained from the data collapse shown in Figs 6 and 7. We also used Γ_c_=0.135 for *N*=1,024, Γ_c_=0.12 for *N*=4,096 and Γ_c_=0.115 for *N*=16,384 that are the values found for the transition to chaos. The maximal cluster size 
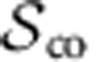
 was assumed to be proportional to a power law of the system size since at the steady state the correlations span the entire system (*ξ*∼*L*):


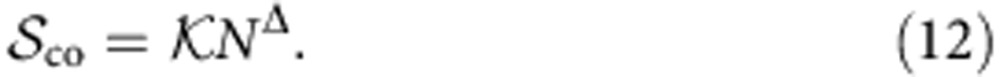


where 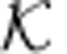 and Δ are constants. We found the parameter values 
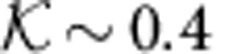
, *A*∼4.547, *B*∼30.51 and Δ∼0.482 by minimizing the normalized *L*_2_ norm of equation (11) with respect to the data from simulations:


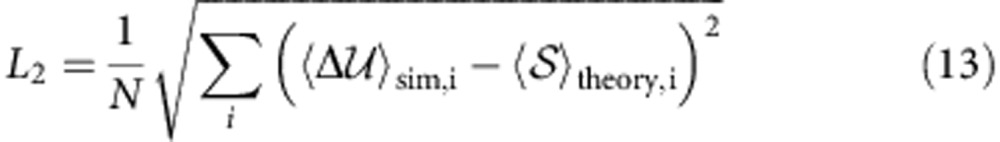


the best fit resulted in *L*_2_∼0.114. Note that the value of Δ∼0.482 is approximately consistent with avalanches concentrated along a shear band and thus proportional to the linear system size *L∼N*^1/2^. Figure 8 shows the first moment of the potential energy fluctuations 
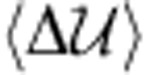
 obtained from the simulations as a function of the maximal strain amplitude Γ, compared with equation (11) for three different system sizes. The most obvious features of 
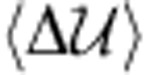
 as a function of the maximal strain amplitudes is the crossover (cusp) in behaviour at the critical point (Figs 8 and 9), which was mentioned above, and the system size-dependent saturation of 
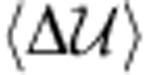
 for very large strain amplitudes. As one can see in the figures, both of these features are described by the theory. The saturation, and dependence on system size can be explained by noting that for very large maximal strain amplitudes Γ→∞, the normalized distribution function converges to the usual power-law statistics 
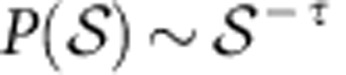
 and, respectively, 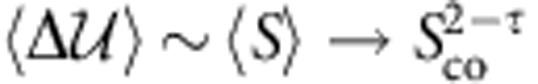. One feature that we observed in the simulations that is not explained by the current theory is that Γ_c_ changes slightly with the strain amplitude due to structural rearrangements. In the theory (equation 1), structural rearrangements will amount to a change in the properties of the distribution of the random pinning *f*_R_(*u*,t). However, this effect is small (changes in Γ_c_ are <5%) and we did not take that into account when fitting the data to the theory. By analysing the avalanche statistics using scaling forms predicted by depinning theory, we have shown that there is a critical point at a critical strain amplitude Γ=Γ_c_, which is the same strain amplitude at which the system undergoes an irreversibility transition. However, this raises the question of why the two occur at the same point. Below we explain this intriguing concurrency.

### Connection between dynamics and critical behaviour

**Figure 10:**
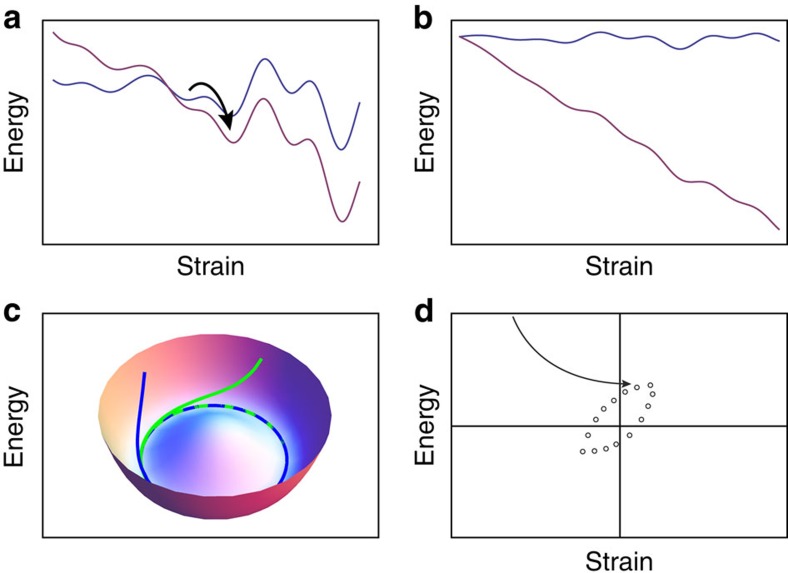
Nonlinear stability. (**a**) Tilted energy landscape—nonlinearly stable. (**b**) Tilted energy landscape—completely unstable. Chaotic behaviour is possible in this scenario. (**c**) A simple example of an attractor: for a dissipative system, different initial conditions that are in the same basin of attraction result in trajectories (blue and green lines), which end-up in the same limit cycle. (**d**) Below the critical strain amplitude, for each strain amplitude, the system finds, after a transient (arrow), a stable configuration (circles).

Here we discuss the connection between depinning and the observed dynamics in the reversible and irreversible regime. The essence of this connection is that at depinning, the external force *F* suppresses all the energy barriers (Fig. 10a,b), which changes the topology of the energy landscape—instead of a set of disconnected energy minima, we have a fully connected set of energy minima in terms of strain. This affects the dynamics and reversibility of the system (a related explanation was suggested for the dynamics of supercooled liquids, see ref. 44).

### Limit cycles

Since the system is dissipative, it will always flow to an attractor occupying a limited part of phase space (Fig. 10c; ref. 45). This attractor will be composed of a finite or infinite set of configurations of the system connected to each other by elastic or plastic displacement (Fig. 10d). For a system under linear shear, when the external forcing is below depinning, it is guaranteed that after some amount of strain the system will find a local minimum of the potential energy (will become pinned). For cyclic strain, if the maximal strain amplitude is below depinning, the system will find, after transient dynamics, a set of configurations all below the critical stress. Since the stress is lower than the critical depinning stress, this set of states is guaranteed to be linearly stable or nonlinearly stable. In the case that is nonlinearly stable, if the stress is increased, the system will overcome a close-by energy barrier but will ‘fall' into an adjunct energy barrier (Fig. 10a), which means that the next configuration in the attractor is separated by a finite energy barrier. Therefore, in this case, the attractor is not chaotic and it must be a limit cycle (periodic). This situation is not so different to an absorbing phase transition, which was suggested as an explanation for similar phenomena^14^,^21^, although we suggest that depinning provides greater physical insight into the reason for the system to reach an absorbing state.

### Chaotic attractor

When the stress is close to depinnig values, a small increase in stress, due to a strain step will overcome all of the energy barriers (Fig. 10b). In this case, the system will be completely unstable, for a short time. In the quasi-static shearing scenario, the system will reach another minimum of the potential energy when the minimization algorithm or dissipation lowers the energy again but before that happens it will spend some time in boundless motion. Since there are effectively no energy barriers in this time, there are no retrieving forces and chaotic motion is possible (in some systems with quenched disorder and with a ‘no passing' property fulfilled^46^, such as charge density waves and certain random magnets, chaotic motion is not possible and there always is a limit cycle^47^, but this is not the case in plasticity in amorphous solids in which disorder is not strictly quenched and for which the no passing rule is broken.).

### Period doubling

When the system is close but still not exactly at criticality, there are less and less stable ‘pinned' configurations. Therefore, the likelihood of the system being able to ‘construct' a limit cycle that returns to the same point after one period is smaller and it may be required to have more than one cycle before the system can return to the initial configuration.

To summarize, if the strain amplitude is below depinning, the system can always self organize into cycles composed of states in which the stress fluctuations never reach depinning values. In that case the dynamics will always be bounded, either linearly or nonlinearly (overcoming one energy barrier). If the strain amplitude is large enough, there are always states in which the stress is very close to depinning. In that case small increase in the stress, due to straining, will generate stresses that are larger than the depinning value, and thus will cause unbounded motion that can lead to sensitivity to initial conditions and chaos.

### Relaxation dynamics

Depinning mean-field theory predicts that close to a depinning transition, the system will ‘slide' for a long time (displace) before it becomes pinned (this is a type of non-equilibrium critical slowing down). Therefore, the accumulated strain to reach a steady state (the number of cycles times 4Γ) is expected to diverge as a function of the applied force:


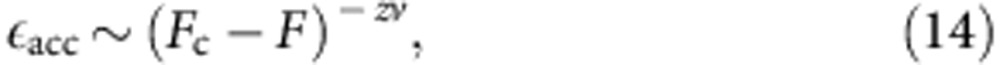


with mean-field depinning theory, which was derived for linear shear, predicting a value of *zv*=1 (ref. 31). Since the steady state is a limit cycle composed of a set of pinned states, we expect that also under oscillatory shear, the accumulated strain to reach a steady state will scale in the same way as the strain needed to pin one state. Since we control the maximal strain amplitude, we obtain on substituting:


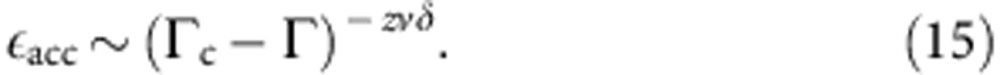


In the simulations, we find power-law scaling with *zv*∼2.4 for a choice Γ_c_=0.11 (Fig. 3d), and *zv*∼1.38 for a slightly smaller Γ_c_=0.1 for the largest system that we studied (*N*=16,384). Note that previously^6^ we calculated the dynamical exponent by considering the number of cycles required to reach a periodic cycle, whereas here we consider the accumulated amount of strain required to reach the limit cycle, which is more compatible with the theory. The dynamical exponent *zv*=1 predicted by mean-field theory is in rough agreement with the scaling of the time to reach steady state measured in the experiments of Nagamanasa *et al.*^14^ on colloidal glasses which gave *zv*∼1.1/*δ*∼0.88.

From the arguments presented above, it is clear why the depinning and irreversibility transitions are inherently connected. Since depinning is a relatively well-studied theory, this connection provides a basis for a deeper understanding of plasticity in amorphous solids.

## Discussion

We have studied the avalanche statistics of amorphous solids under oscillatory shear and have shown that there is a critical maximal strain amplitude at which the correlation length diverges and the avalanche statistics follow theoretical mean-field scaling laws developed for the depinning transition. A key result is that the non-equilibrium critical point occurs at the same maximal strain amplitude as a dynamical reversibility–irreversibility transition, which was recently identified in the same system. We have explained why the depinning transition causes a topological change in the structure of the energy landscape, thereby facilitating a transition from periodic to chaotic dynamics. Furthermore, we have shown that the observed dynamical scaling close to the irreversibility transition is connected to the one obtained by a depinning mean-field theory^31^.

Experiments on shearing of colloidal suspensions^7^,^8^,^14^ have found an irreversibility transition. Similarly, experiments on granular piles have also shown that the onset of irreversible behaviour is associated with the appearance of system spanning events^24^, consistent with our findings. It would be interesting to see if the avalanche statistics that we reported here can also be observed in experiments such as the ones performed by Nagamanasa *et al.*^14^ and Keim *et al.*^8^ on jammed colloidal glasses.

An important issue that was not addressed in our current work is the effect of the dynamics on the structure of the material. For dilute colloidal suspensions^13^ and granular matter^29^, it has been observed that the structure changes during the irreversibility transition. Structural changes in amorphous solids are very subtle and will be harder to observe^48^,^49^,^50^. An implication of the current results is that the onset of plastic failure may be detected from power-law scaling of the power spectra or the slip size distributions of the slip avalanches.

## Methods

### Molecular dynamics simulations

The data used in this work were generated using molecular dynamics simulations of systems of 1,024, 4,096 and 16,384 point particles in two dimensions interacting via an isotropic attractive–repulsive pairwise potential (described in detail in refs 33, 6) where the effective radius of half the particles is 1.4 times larger than the other half. We use the mass *m* of the particles, the typical interaction distance *σ* and the typical interaction energy *ɛ* to define reduced units for the energy (*E*→*E*/*ɛ*), the stress Σ→Σ/(*ɛσ*^−3^), the number density *ρ*→*ρ*/(*σ*^−3^) and the time *t*→*t*/(*ɛm*^−1^*σ*^−2^)^1/2^. Positions of particles are given in terms of *q*→*q*/*L*, where *L* is the system size. The sample is kept at a constant volume and number of particles (NVT) with a number density *ρ*=0.75 in the reduced units that are significantly higher than the jamming transition. Initial frozen amorphous configurations are generated by equilibrating systems of particles at the fluid phase and then quenching them to zero temperature using a minimization algorithm^51^. To verify that the structure is amorphous, we calculated the pair-correlation function. In Supplementary Fig. 1, we show the pair-correlation function for the small particles that shows the typical structure of a frozen liquid and no long-range order. The material is subject to small steps of shear strain (Δ*ɛ*=10^−4^) using the Lees–Edwards boundary conditions^52^. The dynamics under shear is quasi-static (after each shearing step the energy was minimized using the FIRE minimization algorithm^51^). The strain is applied in a periodic manner: first, positive strain steps are applied. When a maximal predecided strain Γ is reached, the strain is reversed by applying strain steps in the opposite direction. This proceeds until the strain reaches the negative value of the maximal strain −Γ. At this point, the strain steps are reversed until the system returns to zero strain, completing the cycle. The cycle is then repeated.

## Additional information

**How to cite this article:** Regev, I. *et al.* Reversibility and criticality in amorphous solids. *Nat. Commun.* 6:8805 doi: 10.1038/ncomms9805 (2015).

## Supplementary Material

Supplementary Information Supplementary Figures 1-6, Supplementary Notes 1-7 and Supplementary References

## Figures and Tables

**Figure 1:**
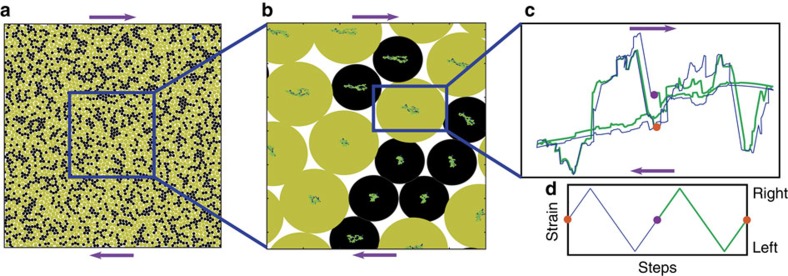
Limit cycles. Repetitive particle trajectories in a period two limit cycle. (**a**) The entire system, (**b**) local environment and the trajectories that each particle is undergoing and (**c**) the trajectory of one particle. (**d**) The strain applied using the Lees–Edwards boundary conditions (purple arrows show how the Lees–Edwards boundaries move with respect to the simulation square when the system is sheared in the positive direction). Since the limit cycle has period two the trajectories repeat themselves only after two shearing cycles (the blue and green lines in **d**). The particle starts from the orange initial point and moves to the right on the blue trajectory, due to the external strain that shears the material to the right, then moves back to the centre and to the left, when the strain is changed accordingly (blue curve on **d**). When the strain is set back to zero, the particle reaches the purple point. Then, when the strain is applied again to the right, the particle moves accordingly, but this time on the green trajectory. The particle then moves to the centre and to the left due to the applied strain (green curve in **d**). Eventually, the particle comes back to the orange point, the initial condition. The same two trajectories repeat in the next two cycles and following cycles.

**Figure 2:**
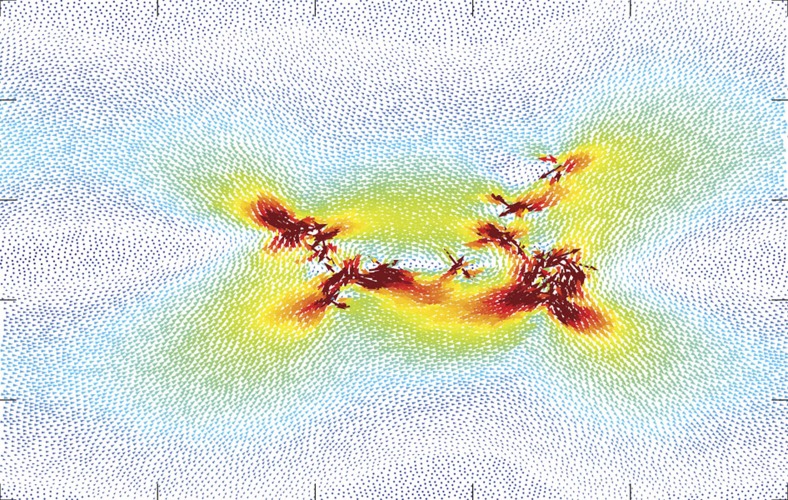
Reversible (repetitive) Avalanches. An avalanche in a subcritical limit cycle for a system with *N*=16,384 particles and maximal strain amplitude Γ=0.1. Even though the avalanche spans a large part of the system, it is repeated under repeating strain cycles of identical strain amplitude. The arrows mark the displacement during the avalanche and the colours represent the magnitude of the displacement (warm—large displacement and cold—small displacement).
