# Supplementary material for: Reversibility and criticality in amorphous solids
Source: Nat Commun. 2015 Nov 13;6:8805. doi: 10.1038/ncomms9805 (PMC4660054; doi:10.1038/ncomms9805)
Supplement: Supplementary Information — Supplementary Figures 1-6, Supplementary Notes 1-7 and Supplementary References [file ncomms9805-s1.pdf]

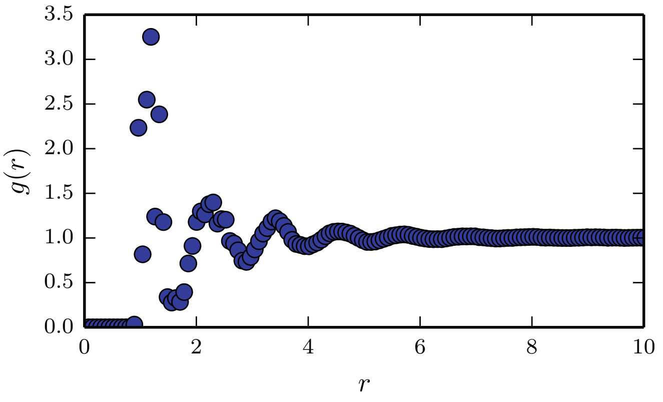

Supplementary Figure 1. Pair correlation function for the initial snapshot.

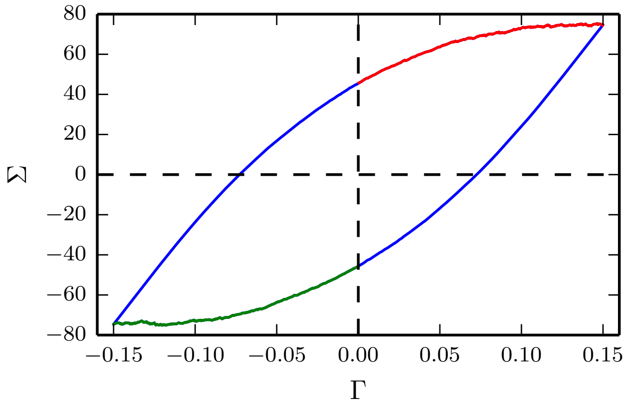

Supplementary Figure 2. Stress-strain curve exhibiting Hysteresis. Red and green branches are the relevant parts of the curve for the avalanche statistics. In the calculation we assume that they provide identical statistics.

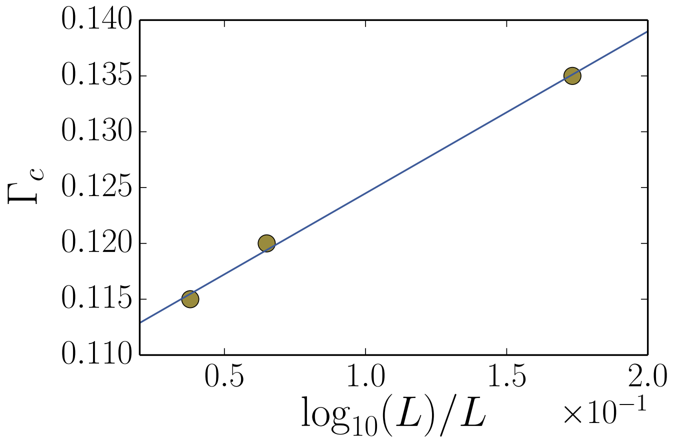

Supplementary Figure 3. Finite size effects in the critical strain amplitude.

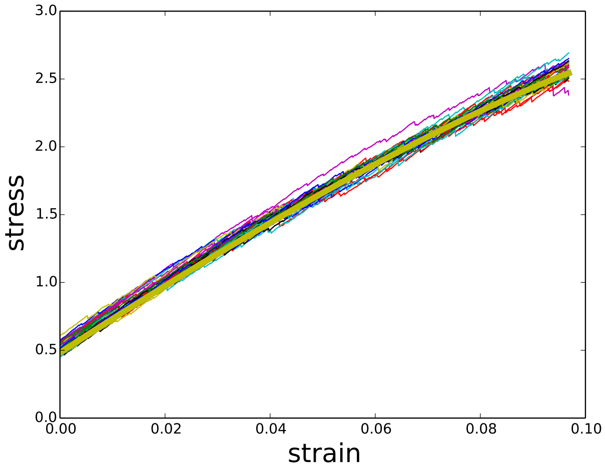

Supplementary Figure 4. Comparison of 30 stress-strain curves from simulations ($N = 16384$, $\Gamma = 0.097$) with equation 5 in the main text, (thick dark-yellow curve) with a critical exponent $\delta = 1.25$.

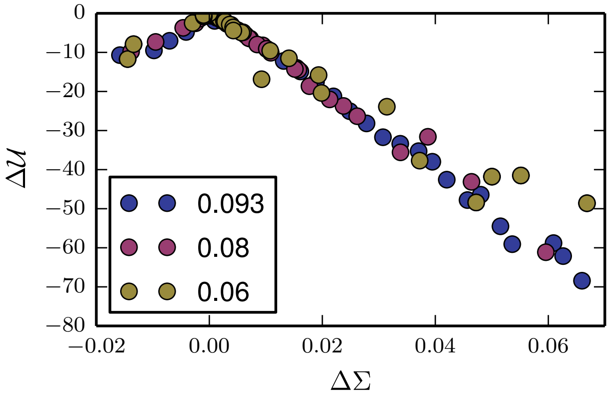

Supplementary Figure 5. Average energy drops vs stress drops for three different maximal strain amplitudes. The figure shows that the energy drops grow linearly with the stress drops.

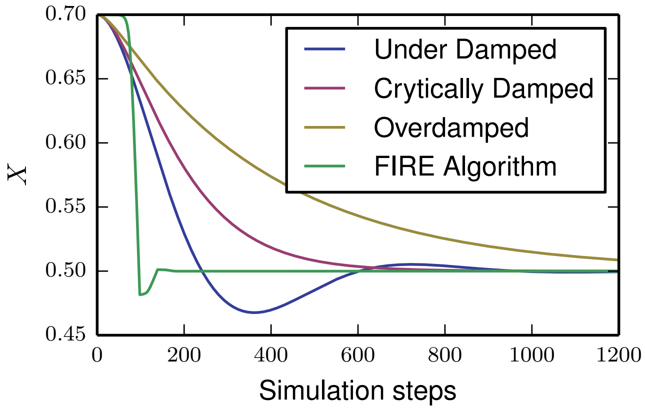

Supplementary Figure 6. Relaxation of the coordinate of an harmonic oscillator with equilibrium position $x = 0.5$ for underdamped, criticallydamped and overdamped dynamics compared with the FIRE algorithm.

Supplementary Note 1: Stress-strain and Hysteresis

One immediate consequence of the existence of avalanches (and plastic events in general) is that the stress strain curve becomes non-linear and exhibits hysteresis - the stress becomes a multivalued function of the strain (see Supplementary Figure 2). In principle, this nonlinearity can be deduced directly from the avalanche statistics. In the case of amorphous plasticity, however, this calculation is too complicated and in order to obtain an accurate scaling law we have to resort to fitting equation 5 in the text:

$$(\Sigma_\mathrm{c} - \Sigma) \sim (\Gamma_\mathrm{c} - \Gamma)^{\delta} \,. \tag{S.1}$$

we fit this relation to stress-strain curves obtained from simulations and get good agreement with $\delta = 1.25$ (see Supplementary Figure 3). This expression describes the upper branch of the hysteresis loop. Since the loop is symmetrical for positive and negative shearing (see Supplementary Figure 2) we always assume that equation 5 describes the forward motion.

Supplementary Note 2: Yield and finite size effects

The increment in plastic displacement due u_p to an infinitesimal change in the force is [1]:

$$du_\mathrm{p} \sim \langle \mathcal{S} \rangle_\mathrm{F} dF \,, \tag{S.2}$$

for a small force increment dF over the current force F (the avalanche size $\mathcal{S}$ is the amount of slip or displacement in an avalanche event and $\langle \mathcal{S} \rangle_F$ is the average avalanche size for a constant applied force F). This can be translated to an equation for the force-displacement behavior, using the theoretical scaling law for $\langle \mathcal{S} \rangle_F$:

$$\frac{du_\mathrm{p}}{dF} = \mathcal{C} \left(\frac{F_\mathrm{c}}{F_\mathrm{c} - F} \right)^{\alpha} = \mathcal{C} f^{-\alpha} \,, \tag{S.3}$$

where $\mathcal{C}$ is a constant with dimensions of length/force, $f = \frac{F_\mathrm{c} - F}{F_\mathrm{c}}$ and α is a critical exponent. When the avalanche size diverges, the behavior will be affected by finite size effects. Since mean-field theory predicts that $\alpha = 1$, the critical displacement will diverge as $L \to \infty$. If we assume that the irreversibility transition under oscillatory shear occurs at the same maximal strain amplitude as the non-equilibrium phase transition under linear shear, the theory may naively appear to be inconsistent with the results obtained by Fiocco et al. [2] which observed that the critical strain amplitude for the irreversibility transition decreases with system size. To see that the theory is in fact consistent with the simulations, we note that in the simulations we controlled the maximal strain amplitude and measured the stress and that both scale differently than the displacement u and force F which were used in the theory. The maximal strain amplitude Γ is related to the maximal displacement by $\Gamma = u/L$. If we integrate equation S.3 directly, we

expect to get $u_{\rm p} \sim \ln L^{-1/\nu}$ when $F \to F_{\rm c}$, where ν is the critical exponent associated with the length-scale. This will give a system size dependence of the plastic critical strain amplitude:

$$\Gamma_{\rm p,c} \sim \frac{\ln L}{L} \,. \tag{S.4}$$

However, the total yield strain is the sum of the elastic $\Gamma_{\rm p,c}$ and the plastic $\Gamma_{\rm e,c}$ yield strains:

$$\Gamma_{\rm c} = \Gamma_{\rm p,c} + \Gamma_{\rm e,c} \sim b\frac{\ln L}{L} + \Sigma_{\rm c}/\mu =_{L\to\infty} \Sigma_{\rm c}/\mu \,, \tag{S.5}$$

where μ is the shear modulus, b is a constant and $\Sigma_{\rm c}$ is the critical stress for depinning ($F = F_{\rm c}$). This prediction is compared in Supplementary Figure 4 to the transition to chaos points obtained from our simulations for three different system sizes. By fitting we can estimate the critical strain to be $\Gamma_{\rm c} \approx 0.11$ for infinite systems. This should also be compared with other theoretical results that predict a yield strain due to the appearance of a system spanning plastic event [3].

Supplementary Note 3: Calculating the scaling function

Starting from:

$$\begin{aligned} P(\mathcal{S},\Gamma) &\sim \int_{-\Gamma}^{\Gamma} d\varepsilon\, \mathcal{S}^{-\tau}\mathcal{D}(\mathcal{S}(\Gamma_{\rm c}-\varepsilon)^{\delta/\sigma}) \\ &= \int_{0}^{\Gamma} d\varepsilon\, \mathcal{S}^{-\tau}\mathcal{D}(\mathcal{S}(\Gamma_{\rm c}-\varepsilon)^{\delta/\sigma}) \\ &+ \int_{-\Gamma}^{0} d\varepsilon\, \mathcal{S}^{-\tau}\mathcal{D}(\mathcal{S}(\Gamma_{\rm c}-\varepsilon)^{\delta/\sigma}). \end{aligned} \tag{S.6}$$

We are making two simplifications: first, we perform the integral only in the forward shearing direction (the red part of the curve in Supplementary Figure 2) since the statistics are symmetric to the shearing direction, second, we neglect the second integral because for strain amplitudes that are away from the critical point (the blue parts of the curve in Supplementary Figure 2) the fluctuations are very small (the distribution function is an exponential). Substituting for $\mathcal{D}(x)$, we get:

$$P(\mathcal{S},\Gamma) \sim \int_{0}^{\Gamma} d\varepsilon\, \mathcal{S}^{-\tau}\mathcal{D}(\mathcal{S}(\Gamma_{\rm c}-\varepsilon)^{\delta/\sigma}) \tag{S.7}$$

$$= \int_{0}^{\Gamma} d\varepsilon\, \mathcal{S}^{-\tau} A e^{-B\mathcal{S}(\Gamma_{\rm c}-\varepsilon)^{\delta/\sigma}} \tag{S.8}$$

substituting

$$x = B\mathcal{S}(\Gamma_{\rm c}-\varepsilon)^{\delta/\sigma} \tag{S.9}$$

$$dx = -\frac{B\mathcal{S}}{\sigma/\delta}(\Gamma_{\rm c}-\varepsilon)^{\delta/\sigma-1}d\varepsilon \tag{S.10}$$

$$d\varepsilon = -\frac{\sigma/\delta}{B\mathcal{S}}(\Gamma_{\rm c}-\varepsilon)^{1-\delta/\sigma}dx \tag{S.11}$$

$$\varepsilon = \Gamma_{\rm c} - \left(\frac{x}{B\mathcal{S}}\right)^{\sigma/\delta} \tag{S.12}$$

$$d\varepsilon = -\sigma/\delta\left(\frac{x^{\sigma/\delta-1}}{(B\mathcal{S})^{\sigma/\delta}}\right)dx \tag{S.13}$$

$$P(\mathcal{S},\Gamma) = -\mathcal{S}^{-\tau-\sigma/\delta}\frac{A\sigma/\delta}{B^{\sigma/\delta}}\int_{B\mathcal{S}(\Gamma_c)^{\delta/\sigma}}^{B\mathcal{S}(\Gamma_c-\Gamma)^{\delta/\sigma}} dx\, x^{\sigma/\delta-1}e^{-x} \tag{S.14}$$

close to the critical point $\Gamma \to \Gamma_c$ and the typical avalanche size $\mathcal{S}$ is very large. Therefore, we approximate the limits:

$$\sim -\mathcal{S}^{-\tau-\sigma/\delta}\frac{A\sigma/\delta}{B^{\sigma/\delta}}\int_{\infty}^{B\mathcal{S}(\Gamma_c-\Gamma)^{\delta/\sigma}} dx\, x^{\sigma/\delta-1}e^{-x} \tag{S.15}$$

and get:

$$\sim \mathcal{S}^{-\tau-\sigma/\delta}\gamma(\sigma/\delta, B\mathcal{S}(\Gamma_c-\Gamma)^{\delta/\sigma}) \tag{S.16}$$

where $\gamma(s,x)$ is the lower incomplete gamma function. This gives the scaling relation:

$$P(\mathcal{S},\Gamma)\mathcal{S}^{\tau+\sigma/\delta} \sim \mathcal{F}\{\mathcal{S}(\Gamma_c-\Gamma)^{\delta/\sigma}\} \tag{S.17}$$

where $\mathcal{F}(x) = -\gamma(\sigma/\delta, -x)$ and $\gamma(a,x)$ is the complementary gamma function.

Supplementary Note 4: Energy vs stress drops

As demonstrated by Lerner et al. [4] and further developed by Salerno et al. [5] when the system is in a steady-state, there is a simple relation between an energy drop and the concurrent stress drop. The relation stems from the fact that at the steady-state, the work done on the system by the straining is balanced by the energy drops. Thus, they got the sum rule:

$$\frac{\langle\Sigma_s\rangle}{4\mu}\sum_i \Delta\Sigma_i = \sum_j \Delta\mathcal{U}_j \tag{S.18}$$

where μ is the shear modulus, $\Delta\Sigma_i$ is a stress drop, $\Delta\mathcal{U}_j$ is an energy drop and we have assumed that there is a well-defined average stress $\langle\Sigma_s\rangle$ at the steady-state. Under oscillatory shear conditions the steady-state stress depends on the strain amplitude, but for a small strain interval this relation should still hold. Therefore, at the steady-state the sum of the energy drops is proportional to the sum of the stress drops. For large avalanches, which are dominant in determining the power-laws, this suggests that individual stress and energy drops are also proportional. This was confirmed in the simulations by Salerno et al. [5]. Indeed, we also observed this behavior for oscillatory shear where the stress and energy drops were found to be proportional (see Supplementary Figure 5).

Supplementary Note 5: Finite size effects in the data collapse

We expect to have data collapse only for intermediate strain amplitudes - for strain amplitudes smaller than $\Gamma = 0.05$ the statistics are not good enough because there are not many energy drops (about 10 per cycle or less). Furthermore, far from the critical point the avalanche statistics is not expected to show the same behavior since the system is far from the singularity. For strain amplitudes that are too close to the critical point, finite size effects dominate. Close to the critical point we typically use the following expression:

$$g(f) = \xi^{\alpha/\nu}g_0(L/\xi) \tag{S.19}$$

where $\xi = f^{-\nu}$ is the correlation length, $f = \frac{F-F_c}{F_c}$ is the rescaled force, $g(x)$ is our scaling function, L is the system size, α and ν are the critical exponents (ν is the critical exponent of the correlation length) and $g_0(x)$ is the finite size scaling function who's properties are:

$$g_0(x) \to x^{\alpha/\nu},\ x \to \infty \tag{S.20}$$

and:

$$g_0(x) \to C,\ x \to 0 \tag{S.21}$$

where C is a constant. Therefore, close to the critical point we get:

$$g(f) \sim L^{\alpha/\nu} \tag{S.22}$$

which means that one cannot use the same function to describe the scaling behavior for maximal strain amplitudes in the intermediate range and close to the transition.

Supplementary Note 6: Inertial effects

A recent study by Salerno et al. [6], has found different critical exponents for overdamped, underdamped and damped dynamics and the value of τ ranged between $\tau = 1$ and $\tau = 1.5$ depending on the dynamics. Since the FIRE (Fast Inertial Relaxation Engine) algorithm uses an inertia-like effect to minimize the energy, it is possible that inertial effects contribute to the deviation from the mean-field theory which was derived for overdamped dynamics. In Supplementary Figure 6 we show the relaxation profile for an overdamped, underdamped and critically damped dynamics applied to a harmonic oscillator compared to the FIRE algorithm. This clearly shows that the relaxation is not typical overdamped but is closer to critically damped dynamics which might by one explanation as for why the exponents are not exactly compatible with mean-field theory.

Supplementary Note 7: Average fluctuations

In order to calculate the average fluctuation size in a cycle we integrate over the same probability distributions but divide by the strain amplitude:

$$\langle \mathcal{S}^n \rangle_\Gamma \sim \int_0^{\mathcal{S}_{\rm co}} d\mathcal{S}\, \mathcal{S}^n \mathcal{S}^{-\tau} \frac{2}{\Gamma} \int_0^{\Gamma} d\varepsilon\, A e^{-B\mathcal{S}(\Gamma-\varepsilon)^{\delta/\sigma}} \tag{S.23}$$

for $\Gamma < \Gamma_{\rm c}$ and:

$$\langle \mathcal{S}^n \rangle_\Gamma \sim \int_0^{\mathcal{S}_{\rm co}} d\mathcal{S}\, \frac{2}{\Gamma} \int_0^{\Gamma_{\rm c}} d\varepsilon\, \mathcal{S}^n \mathcal{S}^{-\tau} A e^{-B\mathcal{S}(\Gamma_{\rm c}-\varepsilon)^{\delta/\sigma}} + \int_0^{\mathcal{S}_{\rm co}} d\mathcal{S} \frac{2}{\Gamma} \int_{\Gamma_{\rm c}}^{\Gamma} \mathcal{S}^n \mathcal{S}^{-\tau} \tag{S.24}$$

for $\Gamma > \Gamma_{\rm c}$. after integration:

$$\langle \mathcal{S}^n \rangle_\Gamma \sim \int_0^{\mathcal{S}_{\rm co}} d\mathcal{S}\, \frac{2}{\Gamma} \int_0^{\Gamma_{\rm c}} d\varepsilon\, \mathcal{S}^n \mathcal{S}^{-\tau} A e^{-B\mathcal{S}(\Gamma_{\rm c}-\varepsilon)^{\delta/\sigma}} + \int_0^{\mathcal{S}_{\rm co}} d\mathcal{S}\, \mathcal{S}^n \mathcal{S}^{-\tau} \frac{(\Gamma - \Gamma_{\rm c})}{\Gamma} \tag{S.25}$$

Supplementary References

[1] K. A. Dahmen, Y. Ben-Zion, and J. T. Uhl, Physical review letters **102**, 175501 (2009).
[2] D. Fiocco, G. Foffi, and S. Sastry, Phys. Rev. E , 020301(R) (2013).
[3] R. Dasgupta, H. Hentschel, and I. Procaccia, Physical Review Letters **109**, 255502 (2012).
[4] E. Lerner and I. Procaccia, Physical Review E **79**, 066109 (2009).
[5] K. M. Salerno and M. O. Robbins, Physical Review E **88**, 062206 (2013).
[6] K. M. Salerno, C. E. Maloney, and M. O. Robbins, Physical review letters **109**, 105703 (2012).
